# Biologic drugs induced vitiligo: case reports and review of literature

**DOI:** 10.3389/fimmu.2024.1455050

**Published:** 2024-12-17

**Authors:** Xinyi Shao, Tingqiao Chen, Xingyu Pan, Shuang Chen, Yangmei Chen, Jin Chen

**Affiliations:** Department of Dermatology, the First Affiliated Hospital of Chongqing Medical University, Chongqing, China

**Keywords:** dupilumab-induced vitiligo, atopic dermatitis, biological therapy, inflammatory skin diseases, cytokine imbalance

## Abstract

Biological drugs are extensively used to treat various inflammatory diseases, including psoriasis, atopic dermatitis (AD), and rheumatoid arthritis. While generally effective and safe, these therapies have been increasingly associated with secondary development of vitiligo, especially with anti-TNF α and anti-IL17 drugs. Dupilumab, an IL-4 receptor alpha antagonist used in moderate to severe AD, rarely induces vitiligo. This study reports two cases of new-onset vitiligo following dupilumab treatment for AD. The first case involves an 80-year-old male who developed vitiligo patches appeared on the chest, back, and lower limbs after 2 months of dupilumab therapy. Despite discontinuation of dupilumab, the vitiligo did not regress. The second case describes a 14-year-old female who experienced depigmentation on her forehead one month into dupilumab treatment, with partial improvement of vitiligo lesions over time despite continued therapy. This phenomenon may be due to dupilumab blocking type 2 inflammation, disrupting normal skin homeostasis, and exacerbating type 1 inflammation. These cases, supplemented with a literature review, highlight the potential for biologic drug-induced vitiligo and underscore the need for awareness of such adverse events in clinical practice. The mechanisms underlying this phenomenon likely involve disruption of the Th1/Th2/Th17 cytokine balance, suggesting that targeted therapies may inadvertently exacerbate type 1 inflammation, leading to vitiligo. With the rising use of biologics, clinicians should carefully consider the risk of vitiligo when prescribing these treatments.

## 1 Introduction

Biological drugs have become a common treatment for many inflammatory conditions due to their effectiveness and high safety profile (1–3). Atopic dermatitis and vitiligo are chronic, inflammatory skin diseases that can significantly impact the quality of life of those affected (4, 5). Dupilumab, a human interleukin-4 receptor alpha antagonist, inhibits IL-4 and IL-3 signaling (6). It is widely used in patients with moderate to severe atopic dermatitis (AD) who do not respond adequately to conventional therapies. Adverse events such as injection-site reactions, paradoxical erythema, ophthalmic complications, and alopecia areata have drawn the attention of dermatologists (7). However, cases of dupilumab-induced vitiligo in patients with atopic dermatitis are rare in existing literature, and there is a lack of cases in Chinese. Here, we present two instances of new-onset vitiligo in Chinese patients with atopic dermatitis who received dupilumab injections for their condition.

## 2 Case reports

### 2.1 Case 1

**Figure 1:**
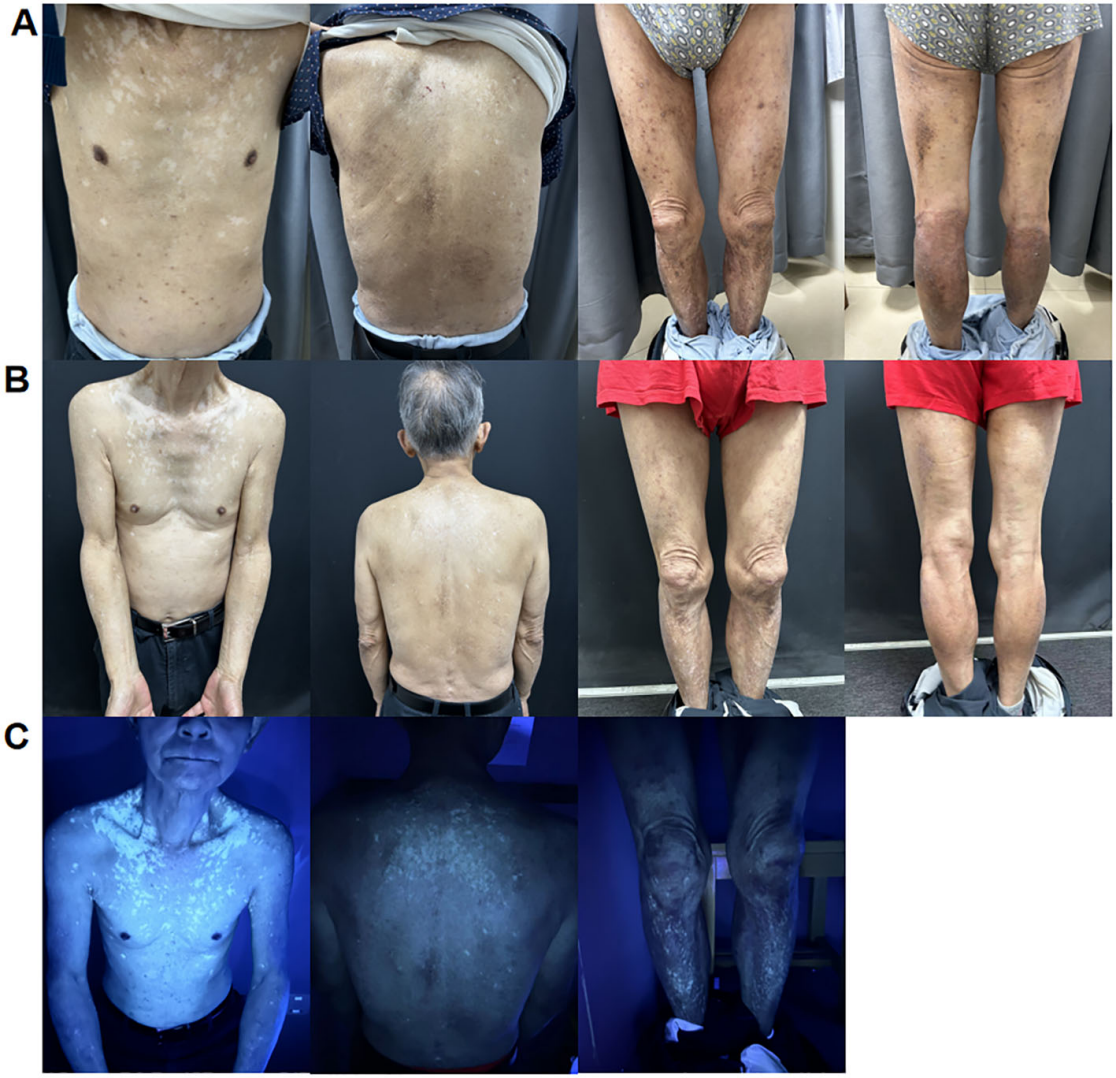
**(A)** Irregular light white patches sparing the chest, back, and lower limb area after 2 months of treatment with dupilumab. **(B)** Further enlargement of white patches on the trunk and lower limbs after 5 months of treatment with dupilumab. **(C)** Woolamp examination of depigmented patches on the chest, back, and lower limbs showing white fluorescence, confirming the diagnosis of vitiligo.

In October 2023, an 80-year-old male patient with AD visited our department due to a poor response to repeated antihistamine treatment and a fungal infection following the use of topical steroids. The patient had a history of AD for more than 30 years; however, he reported no personal or family history of vitiligo. A physical examination revealed erythema, papules, and thickened plaques on the trunk and lower limbs, with a Scoring of Atopic Dermatitis (SCORAD] score: 50.5, an Investigator Global Assessment (IGA] score: 5, and a Numeric Pain Rating Scale (NRS of 10). Laboratory tests revealed an increased serum immunoglobulin E (IgE) level (2990 IU/mL; normal: 5-136 IU/mL). Fungal microscopy confirmed a fungal infection in both lower limbs. According to the typical clinical manifestations of the patient and subsequent auxiliary examinations, AD was identified. Given the poor efficacy of conventional therapies and the presence of a fungal infection, the patient was administered dupilumab (600 mg induction dose and then 300 mg every 2 weeks). After 2 months, there was a slight improvement in erythema and papules (SCORAD: 43.6; IGA: 3; NRS: 4) (**Figure 1A**). However, he developed irregular light white patches on the chest, back, and lower limbs with a Vitiligo Area Score Index (VASI) score of 1.175. The patient requested to continue treatment with dupilumab.

After 5 months of treatment, his erythema and papules essentially vanished (SCORAD: 27.7; IGA: 2; NRS: 2). However, the white patches on his trunk enlarged, and Wood lamp examination revealed bright white patches, indicating that the patient was in the progression stage of vitiligo (VASI: 1.51) (**Figures 1B, C**). It was decided to discontinue dupilumab treatment and use glycyrrhizin. Eight weeks after the cessation of dupilumab, the patches had not reduced in size.

### 2.2 Case 2

A 14-year-old female patient presented with moderate to severe atopic dermatitis, which she had experienced since the age of 4. She had no personal or familial history of vitiligo. Despite treatments with antihistamines and topical corticosteroids, the results were unsatisfactory. Physical examination revealed erythema, papules, and thickened plaques in the hands and both upper limbs, with (SCORAD: 48.4; IGA: 5; NRS: 10). Laboratory assay results revealed increased IgE levels. The patient received dupilumab treatment, receiving an initial loading dose of 600 mg, followed by 300 mg every 2 weeks.

**Figure 2:**
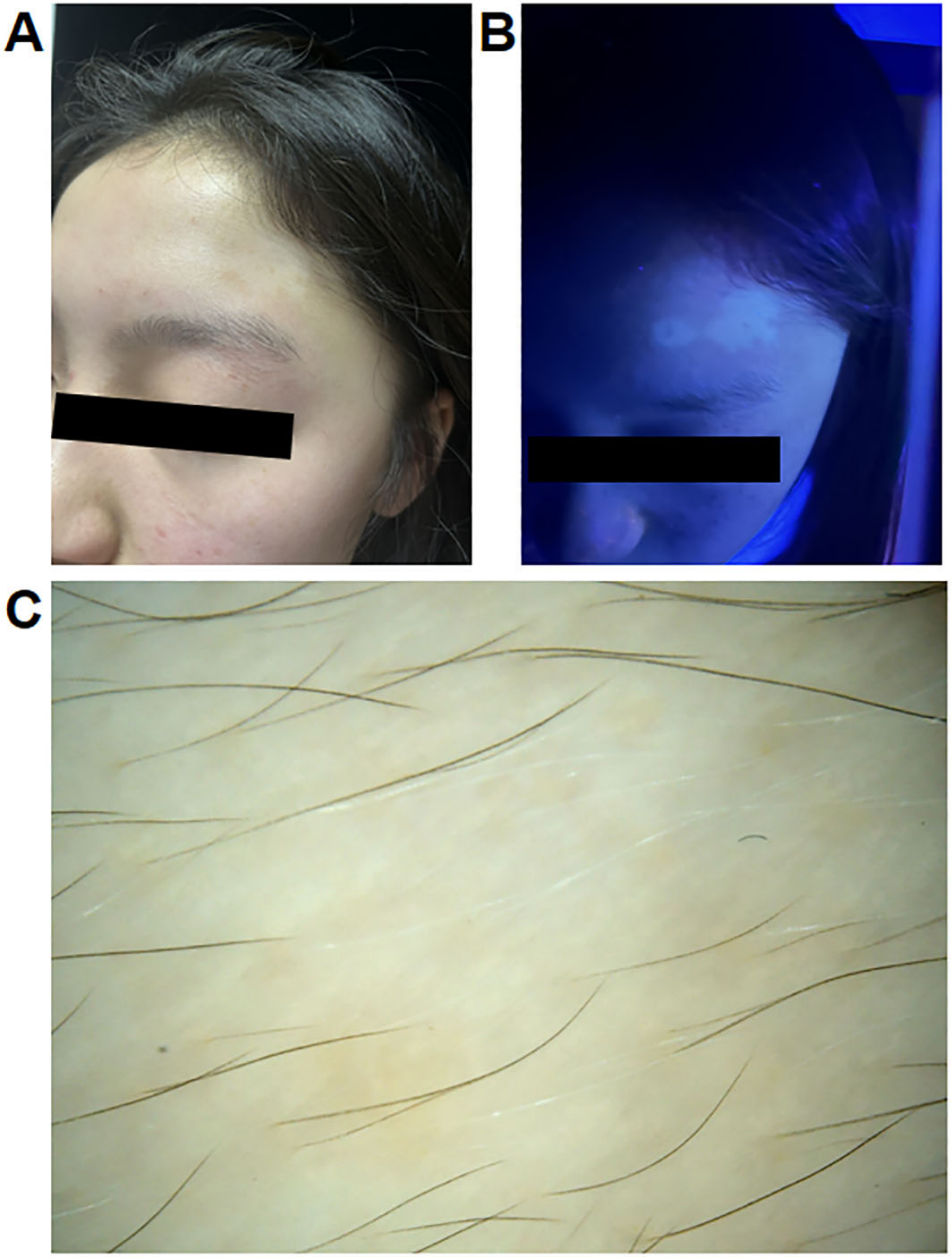
**(A)** Clinical photographs of a 14-year-old female patient with atopic dermatitis who developed vitiligo after treatment with dupilumab. **(B, C)** Wood lamp examination and dermatoscopy of the patient.

At the one-month follow-up visit, improvements in the AD skin lesions were observed (SCORAD: 14.6; IGA: 1; NRS: 3). However, a patch of depigmentation appeared on her forehead, accompanied by gray hair (VASI: 0.24). Dermatoscopy and wood lamp examination confirmed the diagnosis of vitiligo (**Figure 2**). Treatment with topical hydrocortisone butyrate ointment was initiated, and dupilumab therapy was continued at the request of the patient. After five months, partial improvement of the vitiligo lesions was observed.

## 3 Discussion

Here, we present two cases of *de novo* vitiligo, a Th1 cell-mediated inflammatory skin disease, following dupilumab therapy for AD. Vitiligo is a common mucocutaneous depigmentation disorder caused by cytotoxic CD8+ T cell-mediated destruction of melanocytes (5). Recent studies highlighted significant changes in the expression of inflammatory cytokines in vitiligo lesions (8, 9). Consistent with this, previous reports have documented cases of vitiligo induced by biologic drugs such as anti-IL17 agents and anti-TNF α drugs. Despite only two cases of biological agent-induced vitiligo reported in a nationwide cohort study, this adverse event warrants attention. Published cases of biologic drug-induced vitiligo are summarized in **Table 1** (10–38). Thirty-seven cases (26 male patients and 11 female patients) of vitiligo associated with biologic drug use have been reported. Of these cases, 10 were related to adalimumab, nine were related to dupilumab, seven were related to infliximab, five were related to ixekizumab, five were related to secukinumab, and one was related to ustekinumab. Most patients received conventional therapy after developing vitiligo, such as topical steroids, topical tacrolimus and phototherapy. Fourteen patients showed varying degrees of discoloration after treatment, and seven patients showed stable condition of vitiligo after treatment. Notably, six patients chose to continue using biologics, their white patch also improved after treatment.

**Table 1:** Literature review of biologic drugs-induced vitiligo.

Biologics	Year, author	Age (years)	Sex	Biologic drugs indication	Areas Affected	Onset (months)	Biologic drugs withdrawal	Previous history of vitiligo	Management	Outcome
Dupilumab	2021, Takeoka S et al (10)	17	M	AD	Forehead	3	No	Yes	Hydrocortisone butyrate ointment, delgocitinib ointment, 10 sessions of excimer light therapy	After 17 months slight repigmentation, its area has not shrunk.
	2023, Ren H et al (11)	18	M	AD	Bilateral cheeks, neck	3	Yes	No	Tacrolimus 0.1% ointment	N/A
		50	F	AD	Right hairline, neck, and chest	2	Yes	No	Mometasone 0.1% ointment and Nb- UVB	N/A
		40	F	AD	Hands and feet	8	No	No	None	N/A
		50	M	AD	Hands, legs, and feet	1	No	No	Triamcinolone acetonide 0.1% ointment and tacrolimus 0.1%	N/A
		18	F	AD	Pelvis, left leg, and lower torso.	6.25	No	Yes	Triamcinolone acetonide 0.1% ointment	N/A
		43	F	NP	Face, bilateral breasts, chest, abdomen, bilateral forearms and proximal thighs	15	No	Yes	Betamethasone dipropionate 0.05% cream, tacrolimus 0.1% ointment, and Nb- UVB.	N/A
		52	M	NP	Forehead, glabella, nose and cheeks	2	No	Yes	Ruxolitinib 1.5% cream and Nb- UVB	N/A
	2023, Picone V et al (12)	79	M	AD	Scalp, neck, and back of the hands	1	No	No	Topical corticosteroids and nb-UVB	Remission
Ixekizumab	2023, Su HJ et al	72	F	Psoriasis	Face	4	Yes	No	Excimer and cyclosporine	75% repigmentation of the face after 3 months of cyclosporine with control of psoriasis
	2019,Federico Pirro et al (14)	48	M	Psoriasis	Legs, hands, feet	3	No	No	Topical tacrolimus 0.1% ointment for 8 weeks without discontinuation of ixekizumab	50% repigmentation at 4 months of follow-up
	2021, Marasca C et al (15)	53	M	Psoriasis	Face	3	No	No	Topical calcineurin inhibitors	N/A
	2022, Eker H et al (16)	71	M	Psoriasis	Cheeks and perioral areas	2	No	No	Topical corticosteroids and topical pimecrolimus	N/A
	2022, Pathmarajah P et al (17)	36	M	Psoriasis	Trunk, limbs, and face	11	No	No	No	N/A
Secukinumab	2023, Bouzid S et al (18)	30	M	Psoriasis	Hands, palms, and back	9	No	No	Topical steroids	Partial repigmentation
	2020, Nieto-Benito LM et al (19)	39	M	Psoriasis	Axillae, neck, arms, feet	24	No	No	Topical tacrolimus	Stability
	2020, Nieto-Benito LM et al (19)	20	F	Psoriasis	Axillary	12	No	No	Topical tacrolimus	Stability
	2023, Kim JC et al (20)	38	M	Psoriasis	Legs	21	No	Yes	Topical tacrolimus	Stability
	2021, Giordano D et al (21)	42	F	Psoriasis	Upper limbs and trunk	12	No	No	N/A	Repigmentation
Adalimumab	2020, Palazzo G (22)	63	M	Psoriasis	Palms	12	Yes	No	Secukinumab	Resolution
	2020, Tirado-Sánchez A et al (23)	45	M	Psoriasis	Trunk and hands	1	Yes	No	Ustekinumab	N/A
	2020, Phan K et al (24)	24	F	HS	Back and posterior legs	4	N/A	No	N/A	N/A
	2008, Smith DI et al (25)	66	M	Psoriasis	Arms and trunk	5	No	No	N/A	Stability
	2011, Posada C et al (26)	54	F	Crohn’s disease	Upper extremities and trunk	8	No	No	N/A	N/A
	2024, Değirmenci MFK et al (27)	34	M	Idiopathic Uveitis	Lower jaw area	5	No	No	Topical tacrolimus	Stability
	2021, Yang HJ et al (28)	22	M	Healthy volunteer	Neck and chest	2	Yes	No	A combination of weekly excimer laser and twice per day application of topical tacrolimus	Partly repigmentation
	2015, Jung JM et al (29)	39	F	Crohn’s disease	Extremities	12	No	No	Excimer laser and topical tacrolimus	Stability
	2013, Maruthappu T et al (30)	57	M	AS	Face, hands, and axillae	3	Yes	Yes	Topical clobetasol proprionate ointment and tacrolimus 0.1% ointment	Partial repigmentation
	2013, Maruthappu T et al (30)	30	M	AS	Trunk and limbs	6	No	No	N/A	Stability
Ustekinumab	2020, Gedikli OK et al (31)	33	M	PsA	The dorsum of the fingers	4	N/A	No	N/A	N/A
Infliximab	2014, Carvalho CL et al (32)	46	F	RA	Left upper limb and left hemithorax	2	No	No	Topical 10% phenylalanine, clobetasol 0.025%, 2% vitamin E, and Polipodium leucotomos 240 mg/day orally for 30 days.	No improvement
	2005, Ramírez-Hernández M et al. (33)	61	M	RA	The dorsa of the hands	6	No	No	Oral polypodium leucotomus extracts and topical pseudocatalase cream	50% repigmentation
	2013, Mattox AR et al. (34)	60	M	Pityriasis Rubra Pilaris	The dorsal fingers and left wrist	28	Yes	No	Tacrolimus 0.1% ointment twice daily and nb-UVB	Repigmentation
	2017, Ryu TH et al (35)	34	M	Ulcerative colitis	The right mandibular and auricular area	4	N/A	No	N/A	N/A
	2019, Lu X et al (36)	39	M	Psoriasis	Forehead	3	No	No	N/A	Repigmentation
	2011, Ismail WA et al (37)	51	M	Ulcerative colitis	Face	1	Yes	No	Topical tacrolimus ointment, combined with excimer laser	Improvement with the reappearance of pigmentation near the margins
	2017, Luber RP et al (38)	83	M	Crohn’s disease	Limbs and trunk	8	No	No	A combination of compounded tacrolimus 0.1% ointment and calcipotriol–betamethasone dipropionate ointment, both twice daily	Moderate improvement

AD, atopic dermatitis; AS, ankylosing spondylitis; F, female; HS, hidradenitis suppurativa; M, male; nb-UVB, narrow-band ultraviolet B; NP, nasal polyposis; NS, non-segmental; PsA, psoriatic arthritis; RA, rheumatoid arthritis.

N/A, not available.

The mechanisms underlying the development of vitiligo during biological therapy remain unknown. Previous studies suggested that blocking the TNF α pathway may have therapeutic potential in treating vitiligo skin lesions. However, some clinical cases have also reported that TNF α inhibitors may be associated with the emergence or progression of vitiligo (23–38). The underlying mechanism of anti-TNF α agents-induced vitiligo may involve local changes in cytokine balance and the activation of alternative pathways such as type I interferon (39). Additionally, TNF α may decrease regulatory T cell (Treg) production and activation and impair Treg skin homing that allows T cells to self-react to melanocytes (40). IL-17 antagonist-induced vitiligo highlights the delicate balance between Th1 and Th17 regulation. The absence of one of these effector cytokines can promote a response dominated by the other. A previous animal study suggested that IL-17 deficiency contributes to increased IFN-γ+Th1 cells and an elevated Th1 response (41). Additionally, because of the association between the onset of vitiligo and genetic variations, some studies suggested that biologics may cause vitiligo by intervening in the innate immunity of specific genetically susceptible patients (42, 43). Vitiligo is related to other immune diseases; therefore, vitiligo development may be coincidental and related to underlying diseases (40).

Vitiligo and AD are common chronic autoimmune inflammatory skin diseases. Previous studies indicated that patients with AD are at a higher risk of developing autoimmune diseases such as vitiligo. Conversely, individuals with vitiligo have an elevated incidence of AD compared to those without vitiligo (44, 45). The treatment of vitiligo is more difficult than that of AD; however, the use of ruxolitinib may allow repigmentation after vitiligo (46).

Dupilumab is a fully human monoclonal antibody targeting the IL-4 receptor α, inhibiting both IL-4 and IL-13 signaling. Only a few isolated cases of dupilumab-induced vitiligo have been reported. This study contributes to the existing literature by documenting additional cases, specifically highlighting instances of vitiligo induced by dupilumab in Chinese patients. We speculated that this phenomenon occurs due to dupilumab disrupting the balance between helper T cell (Th) 2 and Th1/Th17 pathways. Sushama S et al. suggested that dupilumab-induced IL-4 inhibition leads to Th1/Th17 polarization, resulting in increased expression of IL-17, IL-2, TNF-α, and IFN-γ, which are implicated in the pathogenesis of vitiligo (47). Dupilumab blocks type 2 inflammation, disrupting normal skin homeostasis and potentially exacerbating type 1 inflammation, thereby inducing vitiligo.

With the increasing use of biological agents in patients with skin diseases, clinicians should consider the possibility of adverse reactions like vitiligo when selecting targeted biological therapies. However, our study also has some limitations, including a small sample size and the possibility of potential confounding factors. Future clinical studies with larger sample sizes can better clarify the possible mechanisms of biologics induced vitiligo, and future studies could examine if JAK inhibition may be superior in patients.

## Data Availability

The raw data supporting the conclusions of this article will be made available by the authors, without undue reservation.
